# A Research Agenda for Helminth Diseases of Humans: Social Ecology, Environmental Determinants, and Health Systems

**DOI:** 10.1371/journal.pntd.0001603

**Published:** 2012-04-24

**Authors:** Andrea Gazzinelli, Rodrigo Correa-Oliveira, Guo-Jing Yang, Boakye A. Boatin, Helmut Kloos

**Affiliations:** 1 Escola de Enfermagem, Universidade Federal de Minas Gerais, Belo Horizonte, Brazil; 2 Instituto Nacional de Ciência e Tecnologia em Doenças Tropicais – INCT-DT, Belo Horizonte, Brazil; 3 Centro de Pesquisas Rene Rachou, FIOCRUZ MG, Belo Horizonte, Brazil; 4 Department of Schistosomiasis Control, Jiangsu Institute of Parasitic Diseases, Jiangsu, People's Republic of China; 5 PO Box CT 1380, Accra, Ghana; 6 Department of Epidemiology and Biostatistics, University of California Medical Center, San Francisco, California, United States of America; Ministère de la Santé Publique et de la Lutte contre les Endémies, Niger

## Abstract

In this paper, the Disease Reference Group on Helminth Infections (DRG4), established in 2009 by the Special Programme for Research and Training in Tropical Diseases (TDR), with the mandate to review helminthiases research and identify research priorities and gaps, focuses on the environmental, social, behavioural, and political determinants of human helminth infections and outlines a research and development agenda for the socioeconomic and health systems research required for the development of sustainable control programmes. Using Stockols' social-ecological approach, we describe the role of various social (poverty, policy, stigma, culture, and migration) and environmental determinants (the home environment, water resources development, and climate change) in the perpetuation of helminthic diseases, as well as their impact as contextual factors on health promotion interventions through both the regular and community-based health systems. We examine these interactions in regard to community participation, intersectoral collaboration, gender, and possibilities for upscaling helminthic disease control and elimination programmes within the context of integrated and interdisciplinary approaches. The research agenda summarises major gaps that need to be addressed.

## Introduction

A distinct feature of helminthiasis and other neglected tropical diseases (NTDs; see Box 1 for abbreviations list) is that they occur mostly in poor communities, where conditions for transmission are rife and where they play an important role in contributing to poverty [1]. In this context Hotez and Yamey [2] aptly included the helminth diseases in the group of “neglected infections of poverty”. This classification requires prevention, control, and elimination efforts, considering the wide range of social, cultural, economic, and political factors underlying vulnerabilities and disease risks. The study of these factors has been neglected, and attempts to closely work with other disciplines and sectors have remained largely tokenistic and rhetorical [3]. Furthermore, the characteristic of helminth diseases of having different life cycles, with water, soil, food, and insect vector transmission routes imposes an additional great challenge with significant diversity in disease ecologies that must be considered in the development of interventions to render them effective and sustainable.

**Box 1. List of Abbreviations**

**AIDS**, acquired immunodeficiency syndrome

**APOC**, African Programme for Onchocerciasis Control

**ComDT**, community-directed treatment

**DRG4**, Disease Reference Group on Helminth Infections

**DALY**, disability-adjusted life year

**HIV**, human immunodeficiency virus

**LF**, lymphatic filariasis

**MDA**, mass drug administration

**MDG**, Millennium Development Goal

**NGO**, non-governmental organisation

**NTD**, neglected tropical disease

**STH**, soil-transmitted helminthiasis

**WHO**, World Health Organization

**TDR**, Special Programme for Research and Training in Tropical Diseases

**UN**, United Nations

The study of NTDs is often considered to be a biomedical endeavor centered on chemotherapy, spearheaded by mass drug administration (MDA) [4], [5]. The chemotherapy “tool” is widely considered to be the most appropriate and effective strategy to helminth control in view of the inherent difficulties of implementing alternative control strategies such as a safe water supply and sanitation [6], [7] and the characteristically slow pace of improvement in socioeconomic conditions in resource-poor areas [8]. Although major reductions in the prevalence, intensity, morbidity, and even socioeconomic impacts of helminths have been achieved through chemotherapy, it is generally accepted that improvements in domestic water supplies, environmental sanitation, housing, health education, access to health services for diagnosis and treatment, and vector control must be integrated in control and elimination programs to assure their effectiveness [9], [10]. These goals have not been met in many communities. Although these improvements may not be generalised to all infections in the context of control/elimination, for example access to water and sanitation will not impact on control/elimination efforts for onchocerciasis, they must be seen as important measures to improve the general health conditions of the population. These and other relevant social goals have not been achieved in many communities.

The increasing need to plan, design, implement, monitor, and evaluate programs “outside the box” (beyond the health sector) in an effort to make them more effective and sustainable will require increased engagement of other disciplines, particularly the social sciences, but also the biological sciences and environmental engineering, as well as intersectoral and community-based approaches [3], [11]–[13]. Anthelmintic treatment needs to be seen as a necessary, but not sufficient, condition toward breaking the cycle between helminth infection, illness, and chronic poverty with an important and highly necessary improvement in delivery [14]. Urgency to chart a new course of action using the present and other alternative approaches in helminth disease operational research is further indicated by the increasing concern over the slow progress toward attaining the Millennium Development Goals (MDGs) by 2015 [15], [16]. This situation is due largely to a combination of the persistence and even deepening of poverty in many developing countries (particularly in sub-Saharan Africa, the poorest region), lack of intersectoral collaboration, neglect of social and ecological factors, and funding shortfalls due to political decisions [3], [8], [17].

Although the disability among the bottom billion that results from NTDs, including the helminth diseases, is enormous, they have not received nearly the same attention as three of the highest mortality-causing infectious diseases, HIV/AIDS, malaria, and tuberculosis [18], largely because the helminthiasis are not perceived as major causes of premature death. The sixth MDG specifically mentions HIV/AIDS and malaria as critical targets for sustainable poverty reduction by the year 2015, but merely alludes to chronic parasitic worm infections as “other diseases” [18]. Nevertheless, the morbidity and sequelae they cause are significant. Onchocerciasis can cause visual impairment and blindness; lymphatic filariasis (LF) can cause major body deformation and impaired function, reducing the ability of people to work and look after themselves and others; soil-transmitted helminthiasis (STH) and schistosomiasis can markedly reduce the growth and development of children, including cognitive ability throughout life, and increase child mortality. Hookworm infection often results in anaemia and impacts on maternal health and neonatal mortality. In pregnant women, the STHs may result in premature birth, low birth weight, and increased maternal morbidity and mortality. Liver fluke infections can cause major liver pathology, including hepatic cancer, while neurocysticercosis is a major cause of seizure disorders and other forms of neurological disease, which can increase the level of poverty.

It is clear that these diseases prevent the achievement of the first six MDGs. Therefore, their control with low-cost and cost-effective interventions could be the basis for long-term economic growth and development. The control of helminth diseases would enhance educational achievement, improve nutrition, and enhance growth, which will contribute to the development of human capital, thus impacting mainly the achievement of the first six MDGs [18]. Furthermore, the review by Fincham et al. [19] of 83 papers published between 1995 and 2002 revealed increasing evidence that the relationship of STH and the human immunodeficiency virus (HIV) may have created an opportunity for more rapid infection by the HIV as well as quicker progression to AIDS. Moreover, the efficacy of some vaccines against HIV is likely to be impaired by chronic helminthiasis. Studies in Kenya and several other African countries indicate that individuals infected with *Schistosoma mansoni* and possibly other helminths accelerate HIV disease progression and transmission. [20].

It is increasingly accepted that human ecological and health problems cannot be understood, much less resolved, without adequate understanding of their social, economic, cultural, political, and historical contexts and people-environment relations [21]. The overarching concept of social ecology was further expanded by Stokols [22] to examine the multiple effects and interrelatedness of social elements in a given environment, to include both the social and physical environment in health-related studies. Thus, the environment can function both as a potential source of disease and as a health-enhancing setting providing health resources, including community sanitation systems, health services, and legislation ensuring access to these services. Helminth infection dynamics are the result of the behaviour and livelihoods of individuals in the context of their physical, social, cultural, political, and economic environments. Each of these factors is in a dynamic state of change, evolving over time as they interact with each other, requiring that interventions be contextualised in different socioeconomic, cultural, and geographical settings, which is seldom achieved. Additional needs in addressing the persistence of and vulnerability to helminth diseases include the strengthening of health services and community-level support, which represents a challenge in resource-poor countries and in view of the chronic, non–life threatening nature of helminthiasis, and difficulties in integrating vertical health programs in weak primary care systems [3].

**Figure 1:**
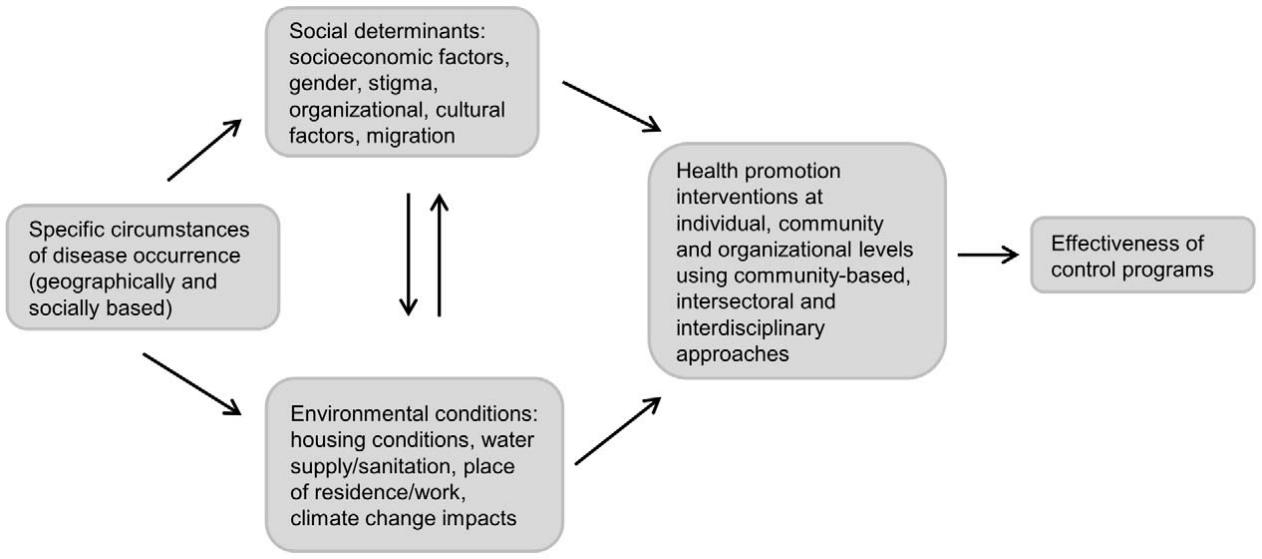
Social-ecological approach to research on social and environmental determinants and health promotion in helminthiasis control. Source: based on [22].

The Disease Reference Group on Helminth Infections (DRG4) has recognised these needs as one of the five umbrella priorities for research and policy for the control and elimination of human helminth diseases. This paper provides an overview of the relations and interactions of these various factors with health resources in the control of the STHs, schistosomiasis, LF, onchocerciasis, food-borne trematodiases, and taeniasis using the strategic social ecological framework developed by Stokols [22] and identifies some research priorities. This paper contextualises the impact of setting-specific and interacting social determinants and environmental factors on the effectiveness of health promoting interventions. The social-ecological approach is particularly well suited to achieve these objectives because social ecology draws on a set of theoretical principles linking diverse personal and environmental factors in human health and disease. Moreover, unlike behavioural change and environmental enhancement strategies, which focus on modifying persons' health-related attitudes, beliefs, and behaviour and improving environmental health parameters, respectively, the social-ecological approach emphasises both. By integrating medical, behavioural and social sciences, and organisational, community development, and regulatory perspectives, the social-ecological approach can facilitate the development of comprehensive and effective health promotion programs [22]. Some of the major components of the social-ecological approach with application to intervention research in the helminth diseases are depicted in Figure 1 and will be elaborated further in this paper.

## Social Determinants

### Poverty

Between 30% and 40% of the estimated 1.4 billion people living in chronic poverty are in sub-Saharan Africa, where helminth disease rates are also the highest [23]. The economic burden of helminth diseases is staggering, but existing data are confined to small-scale studies. Well designed, large-scale studies of their impact on women in particular, the principal food producers and caregivers in most societies, can contribute to a better understanding of productivity losses and therefore promote prevention and control programs [24].

Most of the helminthic diseases we discuss in this paper are closely linked to poverty; they result in part from poverty and contribute to further poverty. The specific and measured effects of the NTDs in terms of the poverty trap have been recently reviewed [25]. They include lower agricultural productivity due to loss of manpower, impairment of childhood education, and treatment costs for people already living in destitution [25].

Lack of capability and rights-based deprivation characterise poor populations such as many rural populations (particularly landless tenant farmers, migrant farm workers, and pastoral nomads), forcefully resettled populations, refugees, and slum dwellers, indicating that many factors are involved in the generation of chronic poverty that will require structural changes in many cases. These and other poor and marginalised populations suffer from under-nutrition, leading to micronutrient deficits, immune deficiencies and, in turn, lower resistance to infection or coinfection with helminths. Moreover, poor individuals, families, and communities characteristically live in degraded and high-risk environments lacking adequate housing, water supply, and sanitation, resulting in close contact with pathogens [16], and have limited access to health services.

### Migrating Populations

Many migrant refugee populations and internally displaced persons, either for political or environmental reasons, and migrant workers are at high risk of contracting helminth infections without adequate access to health care. In 2009, 80% of the estimated 16 million refugees and 26 million internally displaced people worldwide lived in developing countries [26], and in recent years an estimated 100 million migrant workers sent home more than $US300 billion as remittances [27]. Many of them live in camps under crowded, unsanitary conditions, where intestinal parasitoses and malnutrition are highly prevalent, particularly during droughts, food shortages, and armed conflicts [28]. Migrant worker migration has increased and become more complex in the globalised world. Recent trends in migrant labor include increasing sponsorship of migrants by private firms and intermediaries and increasing female migration, resulting in more precarious working conditions and thus increasing health risks [29]. Because of their mobility, many mobile migrants miss treatment by mass chemotherapy and outreach programs [13], an issue that needs further research. Because of this situation, as well as a general absence of screening migrants workers for helminth infections and common lack of clean working environments, and in spite of the existence of a number of international conventions aimed at ensuring the human rights and welfare of migrants, helminth infection rates are high in many migrant groups [5], [30]–[32].

### Social and Health Policies

Although the quality of many social determinants of health is conditioned by public policies and, indirectly by social and macroeconomic policies, relatively few studies have addressed issues of political context, such as the impact of government policies on social inequalities. The benefits of social and public policies is illustrated by the relatively higher social welfare and health levels in some welfare states, such as Kerala in India [33]. Similarly, significant increases in infant vaccination, school attendance, and pre-natal visits to clinics in Brazil were largely attributed to the family assistance programme *Bolsa Familia* (*Family Grant*), particularly in the poor northeastern region [34]. Similar achievements were reported for the *Oportunidades* Program in Mexico [35]. These and other improvements in public welfare and achievements in the control of diseases of poverty require a high level of political commitment within the countries involved. The impact of decentralised and primary health care programmes on the quality and accessibility of diagnostic and treatment services for helminth diseases is an additional issue that needs further study.

There has been increasing recognition that health policy may exacerbate gender inequalities when it fails to address the specific needs of either men or women, and that health systems must address gender equity in order to reduce the health gap between men and women and to improve efficiency. The underlying causes of the gender gap in health include differences between women and men in their health status and health-seeking behaviours, and their access to health care and treatment, all of which affect health outcomes. The consequences of not addressing gender are likely to include persistent excess mortality among women or men in different countries, under-use and inefficient use of health resources, poor user satisfaction and, for some countries, a widening gender gap in health.

### Stigma

While some helminth infections were traditionally neglected as non-life threatening and even considered to be a characteristic of boys growing up, as with urinary schistosomiasis in parts of Africa [36], or necessary for maintaining intestinal health, as with *Ascaris* infections in Ethiopia [37], LF and onchocerciasis are stigmatised in many endemic areas. The characteristic skin conditions in onchocerciasis and the belief that the disease is contagious and can also be transmitted to offspring has resulted in social discrimination, particularly against adolescent girls and young women [38]. Similarly, filarial elephantiasis and hydrocoele in LF were associated with social isolation, emotional distress, and delayed diagnosis and treatment [39], and preference for anonymous health personnel in other communities in northeastern Nigeria reveals an additional stigma-related barrier to health care [40].

### Cultural Factors

The importance of cultural factors in helminthiasis is poorly understood and limited mostly to studies of the use of traditional medicines and illness perception [41]–[43], in spite of the predominance of ethnomedical health systems in many societies. Many ethnomedical health practices and beliefs tend to delay diagnosis and treatment by the regular health services, but recent research also documented positive health outcomes, an area requiring further research [43]. Two beliefs likely to interfere with the treatment for LF are the belief that it is caused by either breaking taboos or witchcraft in northeastern Nigeria [40]. Lower awareness of the causes of helminthiasis, including LF, among poor than more affluent people [44] further adds to their vulnerability. Other areas requiring additional research include the consequences of helminthiasis-linked stigma and people's coping behaviour [45] and the potential value of ethnomedical knowledge systems and household hygiene in public campaigns targeted at helminth diseases [43]. These and many other cultural practices and perceptions point out the need for more culturally competent researchers working in foreign countries and among minority groups [46].

## Environmental Determinants

### The Physical Environment

The natural and human-modified biophysical environment plays a major role in the transmission of helminths, most of which depend for the completion of their life cycles on suitable soil, water, and temperature conditions. At the ecosystem level, biodiversity loss due to the expansion of agriculture, urbanisation, and other human-made environments into natural ecosystems and the creation of new transmission sites have been associated with increasing transmission of vector-borne diseases [47]. Still, longitudinal epidemiological studies of environmental change more specifically related to water resources development and housing conditions need further attention. Alarming increases in the occurrence of LF in urban areas have been associated with stagnant effluent waste water, a preferred habitat of culicidae vectors [48], and with poor housing, but this disease may also be transmitted by anopheline species in water resources development projects [49]. Schistosomiasis risks of hydroelectric and irrigation development projects are well known and attempts have been made for half a century to control the disease. Many of these water development projects are constructed without adequate environmental and health impact assessments and preventive measures and are operated in ways that often facilitate disease transmission. These deficiencies are mostly due to government policies and inadequate intersectoral collaboration [50]–[52]. The Three Gorges dam in The People's Republic of China, which changed the local and downstream hydrology and ecology and resulted in increased transmission of *Schistosoma japonicum* [53], is one of the best known examples of ecological upsets in water resources development.

At the household and peri-domestic levels, unimproved water supplies and environmental sanitation in poor communities are conducive, either directly or indirectly, to the transmission of helminth infections, and the latter also to taeniasis and LF. Poor housing conditions and the spread of polluted surface waters in slums in rapidly growing cities have been associated for several decades with the rapid increase of LF rates in many countries [54], [55].

### Climate Change

There is increasing interest in the impact of climate change on the endemicity of STH and schistosomiasis [56]. The climate change impact has been estimated at 150,000 deaths and 5 million disability-adjusted life years (DALYs) globally over the past 30 years [57], [58], and it is anticipated that increasing temperatures and greater rainfall fluctuations will trigger alterations in physical and biological systems, inducing shifts in the spatio-temporal distribution of disease vectors and intermediate hosts [59], [60]. However, the precise effects of climate change on vector competence, duration of extrinsic incubation periods, survival of vectors, intermediate hosts, and reservoirs, and helminth transmission cycles in general remain poorly understood for the helminthiases [61]. It was postulated that climate change is likely to affect the geographical distribution of freshwater snails, such as *Biomphalaria* spp., an intermediate host of *S. mansoni*. An ongoing water resource project in the People's Republic of China, the “south-to-north water transfer project”, in conjunction with climate change, has probably facilitated introduction of the snail intermediate host of *S. japonicum* (*Oncomelania hupensis*) to new potential habitats in the northern parts of the country [62], [63].

Climate change may also result in a wider range of health risks due to ensuing social and demographic disruptions that swell the stream of environmental refugees [64], challenge the livelihood of poor people, and bring about new challenges for control and changes in access to health services. There are two principal strategies for managing or reducing the risks of environmental change: mitigation and adaptation. The former seeks to reduce the presence and strength of anticipated risk factors (when these are known). The latter accepts that some degree of environmental change is inevitable and seeks to limit its negative impacts by encouraging and investing in preparedness. Large-scale, longitudinal interdisciplinary studies involving the social and biological sciences may inform initiatives and programmes aiming to ameliorate and adapt to climate change impacts [65].

## Health Promotion Interventions

The WHO definition of health systems as a framework comprised of organisations, people, and actions in which people and their institutions play a central role in driving systems [66] and the increasing emphasis on systems thinking [67], are conceptually compatible with the principles of primary health care and the social-ecological approach. Decentralisation of health services can strengthen and facilitate tailoring health resources to local needs through primary health programmes and community health initiatives.

### Mass Drug Administration (MDA)

MDA for helminth diseases is presented, in this collection, in the article by Prichard et al. [68]. However, here we would like to draw attention to the failure of most programmes to examine data on drug uptake in their social, economic, and political contexts. A number of studies in Africa, mostly in Uganda, revealed that the proportion of the population actually taking praziquantel and albendazole tends to be lower than what official reports showed. Factors in these discrepancies included miscommunication between drug distributors and targeted groups, resulting in local resistance to MDA by populations not understanding the rationale for MDA, rumors and conspiracy theories about the “real” objectives of MDA, experiences with adverse events, the hazard of using unpaid volunteers to distribute drugs, and the well known frugality of people living in endemic areas who tend to save drugs for future disease episodes. Moreover, population movements, historical developments, variations in availability of donated relief food, and other factors resulted in sharp variations in drug uptake among districts and over time [13]. The authors concluded that in the case of Ugandan districts, MDA had not generated a demand by 2009 and recommended that health education programmes be upgraded to make treatment more acceptable and that an improved monitoring programme be implemented. They further noted that over-reporting of drug uptake for onchocerciasis and LF in Tanzania has similar root causes as for schistosomiasis and the STH control programmes but called for confirmatory studies. Hotez [6], WHO [32], and Molyneux et al. [69] called for research addressing issues of drug compliance toward achieving optimal effectiveness of MDA.

### Community-Directed and School-Based Treatment

A limitation to the success of control programmes can be the institutional capacity to implement the desired interventions. The acceptability of multiple treatments and the engagement of the community are often overlooked. Community-directed (ComDT) and school-based treatment are cost-effective strategies in helminth disease control and can supplement the low-coverage activities of passive case finding by the regular health services. ComDT may also increase compliance since it is directed to all individuals in the community, involves community residents, and fosters a sense of ownership. These features tend to make the health benefits of integrated interventions more sustainable than those of vertical interventions, for which interest wanes over time. However, ComDT may also be less successful than expected in case of low commitment by community leaders and low priority given to helminth control by communities, or if there is the perception that intervention programmes should be the responsibility of the regulated health services rather than the responsibility of the community [70]. With the increasing use of the unique method of drug delivery of vertical programmes, community-directed intervention, and in some cases the use of common drugs, which is or may be available for other NTDs as well, there is a move from vertical to horizontal approaches in helminth prevention and control programmes.

ComDT was extensively and successfully used in the African Programme for Onchocerciasis Control (APOC), made possible by multi-level public–private partnerships, including the donation of ivermectin. This community-based programme of the APOC subsequently integrated MDA targeting vitamin A deficiency, trachoma, LF, and other parasitic diseases [71]. In the case of both LF elimination and the APOC, interventions rely heavily on ComDT with ivermectin, at least in Africa. One of the challenges faced by these programmes is the issue of inadequate compliance leading to poor treatment coverage in some settings. A crucial social science–driven research to understand “what makes people comply and/or not comply with treatment” will be needed to help redress this phenomenon.

School-based deworming programmes have generally been well received by communities. When combined with health education and food supplementation and in the presence of safe water supplies and sanitation facilities on school compounds, they have significantly reduced helminth infections in many communities and improved general health and nutritional status. In addition, some programmes boosted the educational achievement of children and increased protection from abuse, HIV infection, and dropping out of school. These programmes also help to build effective community partnerships between teachers and health workers, and between the education and health sectors [72]. Treatment of STHs and schistosomiasis reinforced by health education was associated with improved cognition and nutritional status of students in a number of African and Asian countries [73]. Cambodia, which treated its entire school population in 2005, was the first country to reach the goal set by WHO to regularly treat at least 75% of all children at risk of STH and schistosomiasis [74]. In Burkina Faso, the Ministry of Health was able to treat more than 90% of all school children for these two disease groups during one week each, in 2004 and 2005, using exclusively national manpower, a remarkable achievement for a country with the third lowest human development index [75].

ComDT programmes achieved slightly higher treatment coverage than school-based programmes in Uganda and Tanzania, mainly due to the inclusion of non-school children in the former [76], [77]. The relative merits of the school-based and community-based approaches may also depend on initial infection rates, the age of the most at-risk groups, and the phase of programme implementation [77]. A preliminary trial in which Massa et al. [77] were able to develop a platform on the two programmes for vitamin A and bed net distribution suggests that both strategies may be combined to maximise benefits. Such integrated programmes may be embraced if sufficient community resources can be provided and the necessary partnerships with health ministries, the private sector, and non- governmental organisations (NGOs) developed to make them sustainable, although additional studies in different communities are required.

### Health Education

The important role of health education in helminth disease prevention has recently been recognised in the consolidation of the benefits gained by MDA in reducing exposure risk through behavioural change to decrease exposure risk, and in increasing health-seeking behaviour. Health education, when integrated with water supply, sanitation, and housing programmes, can be instrumental in sustaining the benefits of chemotherapy, and preventing infection, as shown above. In populations heavily infected with STH and subject to rapid reinfection, these three preventive measures obtain even greater importance in preventing reinfection. In a school-based deworming campaign in rural Kenya, for example, prevalence of heavy infections decreased only one-third (from 68% to 44.5%) after quarterly treatments over a 3-year period in the absence of health education, suggesting rapid reinfection [78]. Similarly, health education was called for to increase coverage in the administration of albendazole, praziquantel, and ivermectin in a rural area in Ghana [79] and to consolidate gains made with preventive chemotherapy [80]. Innovative health education and communication approaches, including the mass media, can also improve community knowledge about the prevention of the diseases that afflict them, advocate community participation in control programmes, and promote behavioural changes that support interventions [81].

## Upscaling Intervention

### Polyparasitism

Polyparasitism is the result of commonalities in ecological and environmental requirements, infection routes, host exposures, and susceptibility, as well as behavioural, sociological, and economic factors that enable co-occurrence of a multiplicity of parasite–host systems in time and space. As previously mentioned, co-infections have been shown to be a major issue on the outcome of severe diseases. The limited number of studies dealing with coinfections has certainly limited our capacity to develop effective control programmes and may have significant impact on vaccine efficacy as discussed in the various papers in this collection. Coinfection with more than one helminth, as well as other parasites, is often the norm rather than the exception in poor countries of the tropics and sub-tropics. Therefore, polyparasitism must be seen as a major issue in the design and evaluation of control and prevention strategies. This will also require a better understanding of the geographic distribution of helminth infections. There are recently developed spatial tools that can effectively monitor their occurrence and direct control efforts to the most affected populations [82], [83].

This scenario has increased advocacy for an integrated approach to the control of helminthiasis, whereby several drugs for a range of helminth infections are administered in a single programme [69], [84]. Integration into vertical programmes with similar strategies and interventions (LF, onchocerciasis, and STHs) is widely considered to be essential to scale up programmes [85], but the population's acceptability of multiple (simultaneous and staggered) treatments and the degree of community engagement in integrated programmes need to be evaluated. Geostatistical modeling of the often focal and overlapping spatial distribution of individual helminth infections can inform the implementation, monitoring, and evaluation of integrated control programmes [86], [87]. Magalhães et al. [88] carried out the first mapping of the intensity of spatially overlapping helminth infections that may further facilitate planning and evaluation of morbidity control programmes. Wider application of this rapid assessment approach requires further improvement in the quality, coverage, and comparability of epidemiological data and the distribution of vectors and intermediate hosts, as well as stepped up training programmes in the use of spatial tools.

### Environmental Enhancement

Modification of the biophysical and domestic environments, either as part of economic development and urbanisation or through focused activities, can reduce helminth transmission. At the household and community levels, the provision of a safe water supply and improved sanitary facilities can significantly reduce the prevalence of schistosomiasis and soil-transmitted helminth infections [7], [89], and adequate housing can prevent transmission of LF. Moreover, an often overlooked benefit of accessible and safe water supplies is their contribution to reducing the often lengthy trip to distant water sources and thus decreased work load of poor women and girls, freeing up time for activities promoting personal and household health and welfare, all prerequisites for reducing poverty levels.

At the national level, Japan was able to eliminate ascariasis [90] and schistosomiasis [91]. The former was achieved through various intersectoral projects supported by strong government support and the latter through modernisation of agriculture that included the change from water buffalos (the major animal host of the *S. japonicum* parasite) to tractors, environmental modification (elimination of snail habitats by cementing canals and leveling of snail habitats), and urbanisation. Similarly, hookworm infection was sharply reduced in the world's industrialised nations through better sanitation and elimination of hookworm habitats through poverty reduction and (inadvertently) urbanisation [92]. Although these successful control programmes in the industrialised North cannot readily be exported to the developing world, successful control programmes have been carried out in several resource-poor countries, as described in this paper. One example is the case of Puerto Rico, which controlled schistosomiasis through socioeconomic development after termination of its schistosomiasis control programme in 1980 [93].

## Cross-Cutting Issues of Participation, Ownership, Empowerment, Equity, and Gender

### Community Participation

Community participation is considered a key approach to controlling NTDs, improving community health, and ensuring sustainable health development since it is a means of increasing people's autonomy and fostering empowerment in developing countries in terms of accessing health resources [66], [94]. Even though there is wide consensus about the importance of community participation in the control of helminth infections, little attention has been given to the communities' interest in participating in such activities. Similarly, little attention has been paid to the relationship between external motivators and community health workers [3]. Many control programmes are implemented without consultation with the afflicted communities, despite an increasing awareness that proceeding in this way would lead to inequalities being maintained and often exacerbated, due to the expectation that communities should be responsible for the resource deficits of their governments. This may result in poorer communities paying proportionally more for their health care. Moreover, lack of understanding by external programme staff working with communities in the contexts in which programmes are implemented and differences in interests and expectations between community and health service personnel can lead to disagreements that have received little attention by researchers [3].

The importance of adopting sound strategies that empower community members to take relatively simple measures to prevent disease and protect their health, as well as adhere to ComDT, is well described in the literature. What is not clear is which strategy is the most effective to address such needs. It is widely thought that active and broadly based community participation in control programmes can lead to the empowerment of individuals in endemic communities. However, many endemic communities lack institutional systems and structures to encourage people to participate in control strategies, and where they are present, they may not function adequately. As discussed above, one example of a successful strategy used in helminth control programmes is the approach used for ComDT for LF and onchocerciasis control in Africa. One of the challenges faced by these programmes is the issue of adequate compliance and/or non-compliance leading to poor treatment coverage in some settings. Social science research on factors related to treatment compliance may help overcome this problem.

### The Need for Intersectoral and Interdisciplinary Approaches

In addition to the WHO's Global Plan to Combat Neglected Tropical Diseases [30], all eight of the United Nations' (UN) MDGs indicate the importance of intersectoral action beyond the health sector to prevent and control these diseases [95]. Drawing inspiration from the Alma Ata Declaration for Health for All in 1978 and more recent public health strategies, numerous interventions have incorporated intersectoral and integrated approaches in developing countries, with encouraging results. In the early 1980s, Gunatilleke [96] revealed the potential effectiveness of the intersectoral approaches in controlling helminthiasis and other poverty-linked diseases in India, Thailand, Sri Lanka, and Jamaica. More recent intersectoral collaboration has been between health and agriculture departments in vector control activities and between health and education departments in school health programmes, but collaborations with information/communication, water resources, and urban planning institutions and organisations remains largely unexplored. There is growing consensus that integrated packages of multiple interventions need to be upscaled from small projects to broader policy and implementation levels to affect structural change that enables and empowers these and other sectors to play a greater role in helminth disease control [3].

Similarly, there is an urgent need for greater social science participation in helminth disease research in areas of public health that facilitate the translation of biomedical research into policy and public health practice. For example, between 2000 and 2010, less than 1.5% of 4,749 publications on dengue, visceral leishmaniasis, onchocerciasis, and chikungunya were based on social science research and a similar proportion on interdisciplinary biomedical/social science research [10]. This gap is all the more surprising because the social sciences developed a wide range of qualitative and quantitative techniques and research approaches that have been applied to research on health services, community-based prevention, programme implementation, and evaluation [3], [10], [11]. Reidpath et al. [10] calls for research supported by more “sophisticated funders and priority setters” committed to social science and interdisciplinary research to overcome this discrepancy.

### Gender Issues

Studies of gender differences in health and the ability of health care services to meet the needs of women and men have shown variations in terms of life expectancy, risk of morbidity and mortality, health-promoting and health-seeking behaviours, and the utilisation of health care services. There is also increasing evidence demonstrating the importance of a number of different social determinants of health showing a clear interaction with gender inequalities in ways that can magnify the impact on health. Most studies on gender have focused mainly on issues affecting women and less on how gender affects the disease experience of men. It is well known that gender and poverty can lead to gender differences not only in vulnerability to disease and access to quality preventive and curative measures, but also in the experience of the impact of ill health [97].

For the human helminthiases, the link between gender and exposure, risk, susceptibility, disease experience, and outcome has been established through numerous epidemiological studies mainly in schistosomiasis, LF and, onchocerciasis [52], [53], but in-depth behavioural and social studies carried out by medical anthropologists have been confined mostly to schistosomiasis [36] and hookworm infection [98]. The dynamic interaction of gender with other determinants of vulnerability, such as socioeconomic status and age, remain poorly understood for human helminthiasis. Little work has been conducted to apply our current knowledge from gender studies to the development of gender-related policy and practice across all areas of the health sector, including human resources and capacity building. These issues need to be examined within a broader political and environmental context that takes into account issues such as inequality, political instability, violence, displacement, and globalisation.

## A Research Agenda for the Social Ecology of Helminth Infections

In view of the research gaps and priorities for helminth environment and social ecology (Table 1) the DRG4 group agreed on a research and development agenda summarised in Box 2. The research priorities broadly apply to the various helminthiases and are based on the discussions of the DRG4. They include interdisciplinary studies of a wide range of social, cultural, and environmental factors and conditions involved in helminth transmission, spread, persistence, and control, such as the role of poverty, environmental and climatic change, and active and broadly based community participation intersectoral collaboration, and equitable access to health services (Box 3). All of them need to be considered in developing and scaling up acceptable, cost-effective, and sustainable control and elimination programmes.

**Table 1:** Existing Knowledge and Research Needs in the Social Ecology, Environmental Assessment, and Control of Helminth Infections.

Core Theme	What We Know	Research Needs
Social determinants and environmental factors in helminth infections	The relationship between infection rates, socioeconomic status, and behaviour, as well as environmental parameters, is complex but inadequately understood	The relative impact of different integrated control programmes in urban and rural communities and high- and low-prevalence areasLongitudinal epidemiological studies of environmental change (water resources development, climate change, and housing conditions)Evaluation of the intersectoral approach and interdisciplinary studies of integrated control programmesOpportunities for and impacts of increased collaboration between (i) communities, development organisations, and government agencies; (ii) different sectors and academic disciplines; and (iii) between disease–endemic countries – South/South collaboration.
Cost-effectiveness of prevention and control programmes	Estimates of cost and cost-effectiveness are mostly based on a limited number of small-scale studiesInformation gaps hinder the efficient and equitable allocation of health resources	Comparative studies of cost and cost-effectiveness of prevention and control programmes using different strategies in communities with different socioeconomic status and polyparasitism levels
Gender	Exposure risk, infection rates, and health-seeking behaviour vary by gender	Interaction of gender with socioeconomic status, ethnicity, and age in exposure risk and health-seeking behaviourDifferential disease experiences and coping behaviour of men and womenGendered exposure risk, infection rates, and health-seeking behaviour in different socioeconomic and cultural settings
Poor people, migrant populations, other high-risk populations, and pregnant women	Poor people, migrants, and other socioeconomically marginalised groups are particularly vulnerable to helminth infections due to high exposure levels and/or lack of access to health careMigrants and pregnant and breastfeeding women commonly miss treatment by mass chemotherapy and outreach programmesOperational deficiency of programmes in diagnosing and treating pregnant and breastfeeding women	The impact of infected individuals and high-risk groups on maintaining high infection rates in the communityThe epidemiological impact of population movements in the control of helminth polyparasitismThe feasibility and epidemiological impact of increased coverage of high-risk groups and pregnant women
Use of drugs in control and elimination programmes	Certain individuals in a given population consistently reject drug treatment for reasons poorly understood	Examination of the role of (i) disease-related stigma and discrimination; (ii) social, cultural, and geographical barriers; and (iii) programme deficiencies in health-seeking behaviour in general and treatment complianceIncrease coverage of routinely non-compliant individuals
Health education and health promotion in control programmes	Control programmes need an educational component to reduce high-risk behaviours and increase health-seeking behaviourFew studies have assessed the impact of health education/health promotion on infection levels	Evaluation of the cultural acceptability, efficacy and sustainability of different health education and health education/promotion strategies. Analysis of their impact on prevalence and transmission in different population groups and communities using standardised methodology.Evaluation of longitudinal programmes using different strategies, as well as well defined and standardised methodologies
Community participation in prevention and control programmes	Community participation can strengthen prevention and control programmes	Assessment of the community-based approach in communities with different social organisations and socioeconomic levels
Accessibility of diagnostic and treatment services	There is inequity in the accessibility and utilisation of health services for diagnosis and treatment of helminthiasisAccess and use of services are influenced by many individuals, health services, and community-related factors	The relative efficacy, feasibility, and cultural acceptability of school-based, community-directed, and vertical control programmes in rural and urban as well as low- and high-prevalence areas

**Box 2. Summary Points for Research on Social Ecology and Environmental Aspects of Helminthiasis**

- It is increasingly recognised that helminth control programmes need to be multisectoral and interdisciplinary to broaden interventions beyond diagnosis and treatment activities. Such comprehensive programmes can more effectively address social and environmental issues' bearing on the transmission and control of helminths in different social and environmental contexts, increase awareness of their health impacts in the population, increase community participation and control efforts, and mitigate environmental risks.
- A major limitation for implementation of the control and elimination of helminthiasis in endemic countries is the inadequate coverage and sustainability of programmes that are not only due to poverty but also to social, cultural, and political forces that remain understudied.
- Innovative interventions that are community-directed and school-based can increase treatment coverage and compliance, promote behavioural change, and promote community ownership of programmes.
- The unprecedented social and environmental changes the world is experiencing are increasingly influencing patterns of human helminthiases. These changes include population increase, mobility and displacement, changes in rural land use, increasing urbanisation, and climate change.
- In upscaling interventions, identification of social-ecological and environmental factors underlying polyparasitism and the design, implementation, and evaluation of interventions require further efforts in the areas of programme integration and spatial analysis that consider not only prevalence, but also intensity of infection.

**Box 3. Research and Development Agenda for Social Ecology and Environmental Studies in Helminthiasis Transmission and Control**

- Identify social conditions and culturally based factors contributing to the persistence of helminth infections (including polyparasitism) that can inform the development of multisectoral and multi-disciplinary prevention and control programmes.
  - Examine the association between poverty and other socioeconomic parameters, health-seeking behaviour, and helminthiasis.
  - Identify groups vulnerable to helminth infections, such as migrants, refugees, poor farmers and other high-risk occupational groups, landless and homeless people, and slum dwellers.
  - Identify and analyze the impact of culturally based illness perceptions and the use of traditional medicines on the diagnosis and treatment of helminth diseases in modern health facilities.
- Examine the role of environmental and climatic changes in helminth infections, including transmission parameters and vector/intermediate host distributions and ecologies.
  - Identify human resources, institutions, and intersectoral linkages that can strengthen public health systems to prevent and that may mitigate health effects of environmental and climate change among vulnerable populations such as poor farmers and environmental refugees.
  - Evaluate the impact of water resources and urban development, agricultural projects, and housing on the transmission and vulnerability to helminths.
- Promote commitment to and upscaling and sustainability of control programmes and integrated helminth control:
  - Evaluate community priorities, attitudes, knowledge, and participation in prevention and control programmes.
  - Determine the operational feasibility and cost-effectiveness of integrating different control programmes for polyparasitism.
  - Assess the contribution of systematic non-compliant persons as well as pregnant/lactating women and under five-year-olds to the maintenance of transmission and study the possibilities of including the former in control programmes.
  - Develop operational research protocols to determine the most effective, cost-effective, and sustainable implementation strategies for providing multiple drug therapies at the population level.
- Develop strategies that promote community participation, and a sense of ownership, as well as social and gender equity towards providing accessible health service:
  - Understand the relationship between communities and their governments that bear on the provision of health and other services compliance, and community participation in helminth control programmes.
  - Evaluate the cost-effectiveness, impediments, and sustainability of community-directed interventions and school-based deworming programmes.
  - Investigate the role of gender toward strengthening the delivery of programmes and improving accessibility and utilisation of services.
  - Evaluate effectiveness and shortcomings of disease- and culture-specific health education programmes and promote standardisation of methods to facilitate comparison of the effectiveness of programmes.
  - Investigate how access to health services is conditioned by poverty and social inequity, and shaped by political and economic factors including health policy and the impact of social assistance programmes.

## Concluding Remarks

This paper summarises the role of social and environmental factors in the transmission, spread, perpetuation, and control of the major helminthiases. The complexities of their interactions and the fact that these diseases are both driven by and caused by poverty demonstrate the need for stepped up efforts to control the helminthiases using interdisciplinary, intersectoral, and community-based approaches involving the health and non-health sectors. Socio-ecological and environmental research has made significant contributions to a better understanding of the conditions promoting helminth transmission and perpetuation, as well as their prevention and control, and several helminth diseases have been reduced or eliminated in a number of countries, further increasing the urgency to use innovative approaches in interventions. Similarities in the ecologies of the major helminthiases and the widespread occurrence of polyparasitism makes the development of integrated approaches aimed at improving the daily lives of neglected populations and their environment particularly compelling. The research strategy presented here considers these similarities but needs to be modified to adapt it to different mixes of helminthiases and environmental and cultural settings and to consider national policies, priorities, resources, constraints, and specific research gaps in different countries and communities.

Additional studies are required to establish to what extent comprehensive, broadly based intervention strategies complement any structural changes resulting from public and social policies dealing with living standard improvements, gender equity, medical services, education, and social welfare, which are required to eliminate the social and environmental conditions commonly associated with the transmission of the helminthiases. A better understanding of the relative contribution and interactions, including synergisms, as well as constraints of interventions using different approaches applied at the national, district, and community levels can inform planners and decision-makers on how best to upscale and optimise programmes. There is much evidence that poverty, the most important social determinant in helminth diseases, can be effectively reduced through a combination of poverty-alleviation programmes and access to affordable health services.
